# Effect of HFE Gene H63D and S65C Mutations on the Severity of Secondary Iron Overload in Children With β-Thalassemia Major

**DOI:** 10.7759/cureus.115199

**Published:** 2026-08-25

**Authors:** Safia K Ali, Elshazali W Ali

**Affiliations:** 1 Department of Hematology, Faculty of Medical Laboratory Sciences, Omdurman Islamic University, Khartoum, SDN; 2 Department of Hematology, Faculty of Medical Laboratory Sciences, Al Neelain University, Khartoum, SDN; 3 Department of Medical Laboratory Sciences, College of Applied Medical Sciences, University of Bisha, Bisha, SAU

**Keywords:** h63d mutations, hfe gene, iron overload, s65c mutation, serum ferritin, β-thalassemia major

## Abstract

Introduction: Coinheritance of *HFE* gene mutations in patients with β-thalassemia major (βTM) may further exacerbate systemic iron accumulation, potentially leading to earlier onset of iron-related complications. This study aimed to screen for *HFE* gene H63D and S65C mutations in children with βTM and evaluate their impact on the severity of secondary iron overload (IO). Materials and methods: This cross-sectional study included 76 Sudanese children with βTM who were all receiving regular blood transfusions and iron chelation therapy. Polymerase chain reaction and Sanger DNA sequencing were used to screen for H63D and S65C mutations. IO severity was assessed by measuring serum ferritin (SF) levels using electrochemiluminescence immunoassay. Results: The H63D mutation was identified in 17 (22.4%) of the thalassemic children; 16 (21.1%) were heterozygous, and one (1.3%) was homozygous. The S65C mutation was not present in any of the participants. SF levels were higher among carriers of the H63D variant allele (CG + GG) than among those with the wild-type allele (CC). Still, the difference was not statistically significant (median: 2051.0 vs. 1460.0 μg/L, p = 0.09). Furthermore, no statistically significant association was observed between the H63D mutation and the risk of myocardial iron loading (p = 0.087). The results revealed a statistically significant positive correlation between SF levels and the total number of blood transfusions (Spearman’s ρ = 0.496, p < 0.001). A weak, positive, insignificant correlation was observed between SF levels and both the time since the last dose of iron chelation therapy (ρ = 0.257, p = 0.100) and C-reactive protein levels (ρ = 0.129, p = 0.267). Conclusions: The frequency of the *HFE* gene H63D mutation was relatively high among Sudanese children with βTM, whereas the S65C variant was absent. Although SF levels were higher in the carriers of the H63D variant allele compared to those with the wild-type allele, the difference was not statistically significant. These findings indicate limited clinical utility for routine screening for the *HFE* H63D and S65C mutations and emphasize the greater importance of optimizing transfusion and iron-chelating therapy practices to manage IO effectively.

## Introduction

β-thalassemia major (βTM) is a severe clinical phenotype resulting from the inheritance of two β-thalassemia alleles, leading to transfusion-dependent hemolytic anemia. Patients with βTM need lifelong red blood cell transfusions and iron chelation therapy to prevent complications [1,2]. While iron overload (IO) in βTM is primarily exacerbated by transfusion therapy, patients with thalassemia can also develop IO even without erythrocyte transfusions [3,4]. The buildup and deposition of iron in tissues increase oxidative stress and cause cellular damage, impairing the function of affected organs and resulting in conditions such as liver cirrhosis, hepatocellular carcinoma, diabetes, cardiac failure, and skin hypopigmentation [5].

The most common form of hereditary hemochromatosis (HH) results from mutations in the *HFE* gene, which interfere with the hepatocyte iron-sensing mechanism. This leads to decreased hepcidin production, increased dietary iron absorption, and iron accumulation in multiple tissues [6]. *HFE*-related HH is a multigenic disorder that can involve several genetic loci, including *HFE-1* with C282Y, H63D, and S65C mutations at 6p21 [7,8]. It is the most common genetic disorder among people of European descent, and if not treated, it can lead to serious health problems and death [9].

The H63D missense mutation in the *HFE* gene is relatively common. It occurs within exon 2 of the *HFE* gene, where aspartate (D) replaces histidine (H) at amino acid position 63, due to a cytosine (C)-to-guanine (G) substitution at position 187 [10]. Depending on the ethnic background, the H63D mutation accounts for 40-70% of hemochromatosis cases that are not caused by the common *HFE* C282Y mutation [11].

However, it is associated with a milder form of the disease, leading only to a partial decrease in the *HFE* protein's ability to reduce the transferrin receptor's affinity for transferrin, and it usually requires compound heterozygosity to be considered a risk factor for HH [12-14].

The S65C mutation is less frequent than H63D, with a heterozygote frequency of about 2% among whites. It results from the substitution of cysteine (C) for serine (S) at position 65, caused by an adenine (A)-to-thymine (T) transversion at position 193 of the *HFE* gene. The clinical significance of the S65C mutation remains debated, as it may cause increased serum iron and ferritin levels but has not been associated with excess tissue iron stores [6,11,14].

Previous studies have reported conflicting results regarding the effect of *HFE* gene mutations on serum ferritin (SF) levels in βTM patients [15-17]. This study aimed to screen for *HFE* gene H63D and S65C mutations in Sudanese children with βTM and to assess their impact on the severity of secondary IO, hypothesizing that *HFE* H63D and S65C mutations may contribute to increased iron accumulation in children with βTM and therefore be associated with higher SF levels compared with non-carriers. By studying these variants in the Sudanese population, this work builds on previous research to determine whether findings from other regions are relevant to this unique clinical and genetic setting.

## Materials and methods

Study design and setting

This hospital-based cross-sectional study was conducted at major hematology clinics across various hospitals in Khartoum State, Sudan, from September 2017 to March 2020. The study population comprised children with βTM who were receiving regular blood transfusions and iron chelation therapy.

Sample size

Because βTM is relatively uncommon in Sudan, a total of 76 children were invited to participate in the study using a convenience sampling technique; they represented all the children with a confirmed diagnosis of βTM who were registered and receiving follow-up care at the major hematology clinics in hospitals in Khartoum State, including Jafer Ebnaof Hospital, Fath Elrahman Elbashir Center, Elbolk Hospital, Ibrahim Malek Hospital, and Bashair Hospital.

Blood sample collection

Blood samples were collected from all participants by an experienced phlebotomist under standard conditions and transferred into both plain blood tubes for biochemical analysis and ethylenediaminetetraacetic acid tubes for molecular analysis.

Biochemical analysis

The primary outcome in this study was SF level, a quantitative marker of IO, to assess whether polymorphic genotypes are associated with differences in iron burden among the study participants. SF was measured by electrochemiluminescence immunoassay using a Cobas e411 automated analyzer and the Elecsys Ferritin kit (Roche Diagnostics International Ltd., Switzerland). CRP was measured using the Cobas c 311 automated analyzer (Roche/Hitachi, Switzerland) with the CRP Latex kit (CRPLX) (Roche Diagnostics International Ltd., Switzerland) to account for the well-known effect of inflammation on SF levels.

Molecular analysis

DNA Extraction

Genomic DNA was extracted from peripheral blood using the innuPREP Blood DNA kit (Analytik Jena, Germany) and stored at −20°C for further analysis.

Polymerase Chain Reaction (PCR)

A PCR reaction mixture was prepared for each sample, consisting of 4 μL of a ready-to-load master mix (Intron Biotechnology, South Korea), 0.8 μl of each of the forward (5’-ACATGGTTAAGGCCTGTTGC-‘3) and reverse (5’-GCCACATCTGGCTTGAAATT-’3) primers (Intron Biotechnology, South Korea), 2 μL of genomic DNA, and 15 μL of distilled water. The mixture was amplified in a thermocycler (Biometra TADVANCED, Germany) under the following conditions: an initial denaturation at 95°C for 2 minutes, 35 cycles of denaturation at 95°C for 30 seconds, annealing at 63.3°C for 30 seconds, and extension at 72°C for 30 seconds, followed by a final extension at 72°C for five minutes.

 Agarose Gel Electrophoresis

The PCR products (4 μl) were separated on an ethidium bromide-stained agarose gel (1.5%) and visualized using a gel documentation system (Biometra BDA compact, Germany). The optimal product size (207 bp) was determined by comparison with a 100 bp DNA ladder.

DNA Sequencing

The *HFE* gene H63D and S65C mutations were analyzed using Sanger sequencing (BGI Genomics, China).

Ethical considerations

This study was approved by the Ethics Committee of the Ministry of Health, Khartoum State, Sudan, and informed consent was obtained from the children's parents before sample collection. All procedures were performed in accordance with the national guidelines for the ethical conduct of research involving human subjects (2008) and the Declaration of Helsinki.

Data collection and analysis

Patient data were collected from medical records and analyzed using SPSS Statistics version 27.0 (IBM Corp. Released 2020. IBM SPSS Statistics for Windows, Version 27.0. Armonk, NY). Skewness and kurtosis were used to assess the normality of continuous variables. SF levels were not normally distributed and were therefore summarized as median and interquartile range (IQR). Categorical variables were presented as frequencies and percentages. Spearman's rank correlation analysis was used to investigate the correlation between SF levels and the number of blood transfusions, the time since the last chelation therapy dose, and CRP levels.

To evaluate the association between H63D genotypes and SF levels, genotypes were categorized into two groups: wild-type homozygotes (CC) and variant-allele carriers (CG + GG). Differences in SF levels between the two groups were assessed using the Mann-Whitney U test. The association between H63D genotypes and risk of myocardial iron loading was analyzed using Fisher's exact test. All statistical tests were two-sided, and a p-value <0.05 was considered statistically significant.

## Results

This study included 76 children with βTM, of whom 46 (60.5%) were males and 30 (39.5%) were females; their mean age was 6.7 years. All participants were receiving regular blood transfusions and iron chelation therapy, but the average time since the last chelation dose was highly variable (mean ± SD: 192 ± 231 days).

The results revealed a statistically significant positive correlation between SF levels and the total number of blood transfusions. A weak, positive correlation was observed between SF levels and both the time since the last dose of iron chelation therapy and CRP levels, but neither correlation was statistically significant (Table 1).

**Table 1:** Correlation of SF levels with the number of blood transfusions, time since the last chelation dose, and CRP level Correlations were assessed using Spearman's rank correlation analysis. Data are presented as Spearman's rank correlation coefficient (ρ) and corresponding p-values. Statistical significance was defined as p < 0.05. CRP: C-reactive protein, SF: serum ferritin

Variable	Spearman’s ρ	p-value
Number of blood transfusions	0.496	<0.001
Time since the last dose of chelation therapy (days)	0.257	0.100
CRP level (mg/L)	0.129	0.267

**Figure 1:**
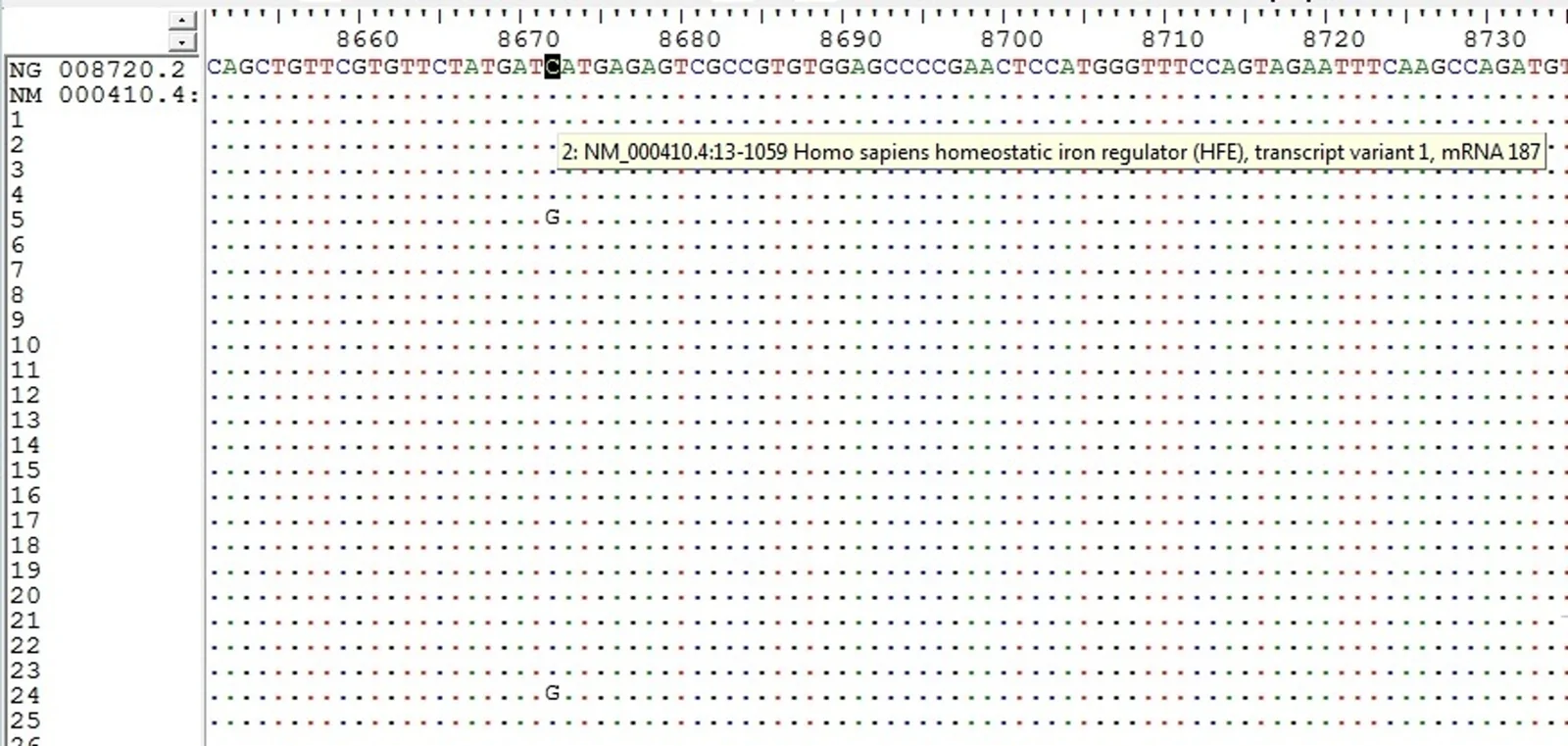
BioEdit alignment Cytosine nucleotide (C) at position 187 (highlighted in black) was substituted by guanine (G).

**Figure 2:**
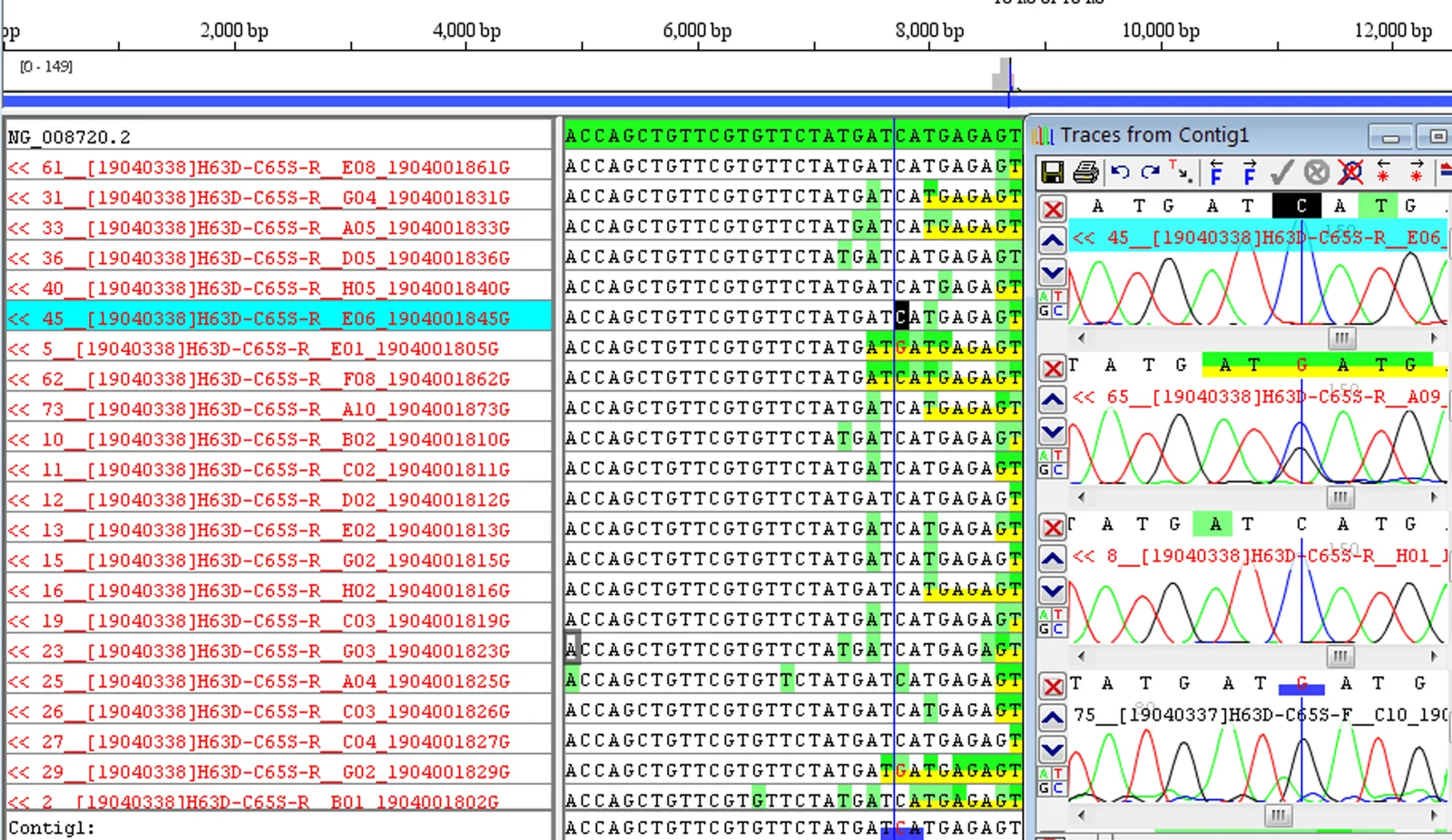
Chromatogram of the H63D mutation The left panel shows the patient accession numbers, and the middle panel shows the alignment, where the substitution of nucleotide C (highlighted in a black box) by G (indicated in red) is observed in patients with serial numbers 5 and 29. The chromatogram on the right shows double peaks: C in blue and G in black, indicating a heterozygous H63D mutation, while a single peak in black indicates a homozygous H63D mutation.

**Figure 3:**
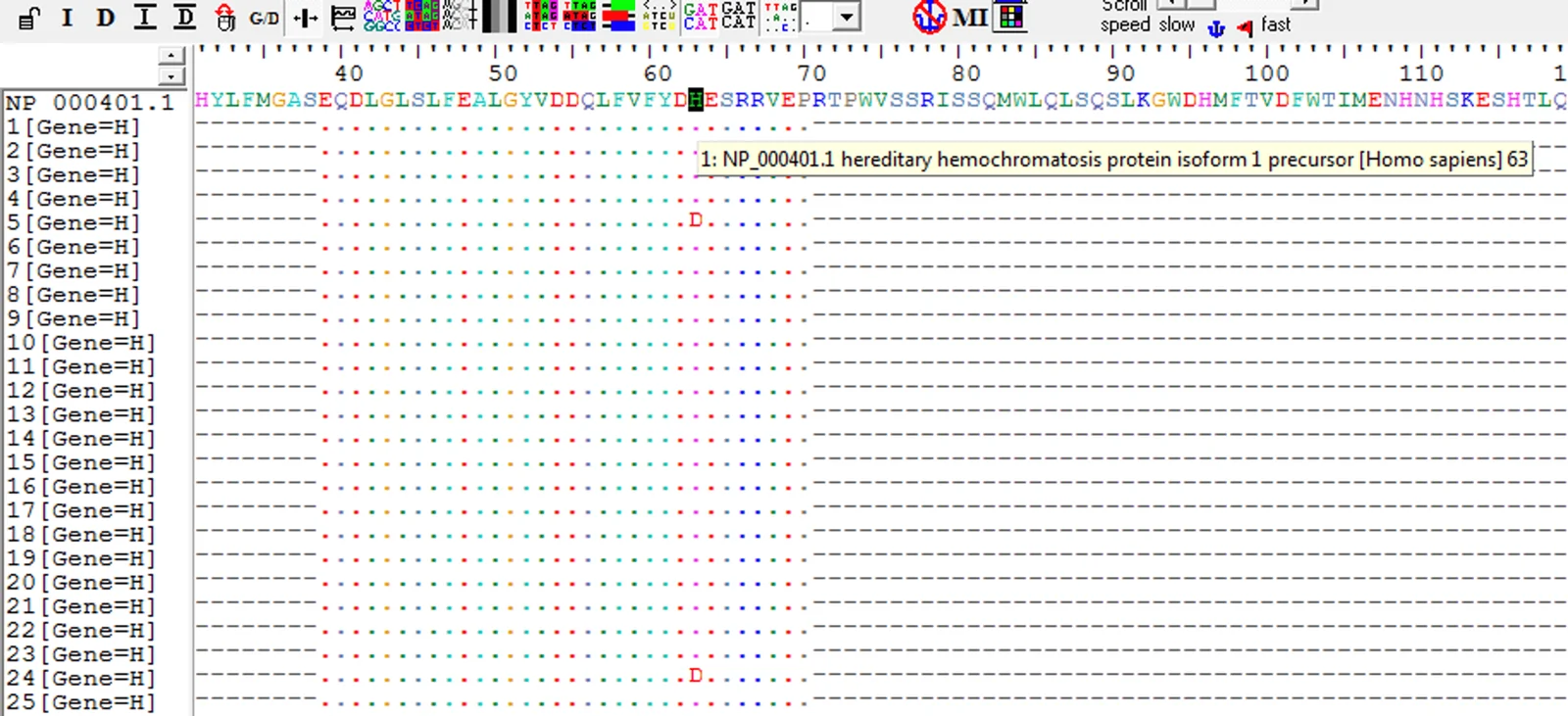
Protein structure change in the H63D mutation The structural model shows the substitution of histidine (H) at position 63, highlighted in a black box, by aspartate (D), marked in red.

As shown in Figures 1-3, sequencing of the amplified fragment of the *HFE* gene for detection of the H63D mutation (rs1799945) revealed the presence of the mutant allele (G) in 17 (22.4%) of the patients; 16 (21.1%) of them were heterozygous (CG), and 1 (1.3%) was homozygous (GG). The frequencies of the wild-type (C) and the mutant (G) alleles were 0.88 and 0.12, respectively.

Although carriers of the H63D variant allele (CG + GG) had higher SF levels than those with the wild-type homozygous genotype (CC), the difference was not statistically significant. As ferritin is an acute-phase reactant, patients with high CRP (>10 mg/L) were excluded from the sensitivity analysis to minimize the confounding effect of inflammation on SF levels. However, the comparison of SF levels between H63D genotypes, after excluding patients with high CRP, remained not statistically significant (Table 2).

**Table 2:** Comparison of SF levels before and after exclusion of patients with high CRP Data are presented as the median (IQR). Comparisons between genotype groups (CC vs CG + GG) were performed using the Mann-Whitney U test. Statistical significance was defined as p < 0.05. CRP: C-reactive protein, IQR: interquartile range

Status	Genotypes	N	Median	IQR	p-value
Before excluding patients with high CRP	CC	59	1460.0	1360.0	0.09
	CG + GG	17	2051.0	2431.0	
After excluding patients with high CRP	CC	31	1360.0	1548.0	1.00
	CG + GG	11	2020.0	3086.0	

Patients were classified into two groups based on SF levels: group 1 (78.9%) with SF <2500 μg/L, considered at low risk of myocardial iron loading, and group 2 (21.1%) with SF ≥2500 μg/L, considered at high risk. The association between the H63D mutation and the risk of myocardial iron loading was not statistically significant (Table 3).

**Table 3:** Association between H63D genotypes and risk of myocardial iron loading Data are presented as numbers and percentages. The association between genotypes and risk of myocardial iron loading was assessed using the Fisher-Freeman-Halton exact test. Statistical significance was defined as p < 0.05.

Genotype	Risk of myocardial iron loading		p-value
	Yes	No	
Wild-type (CC)	10 (16.9%)	49 (83.1%)	0.087
Mutant heterozygous (CG)	5 (31.25%)	11 (68.75%)	
Mutant homozygous (GG)	1 (100%)	0 (0%)	

**Figure 4:**
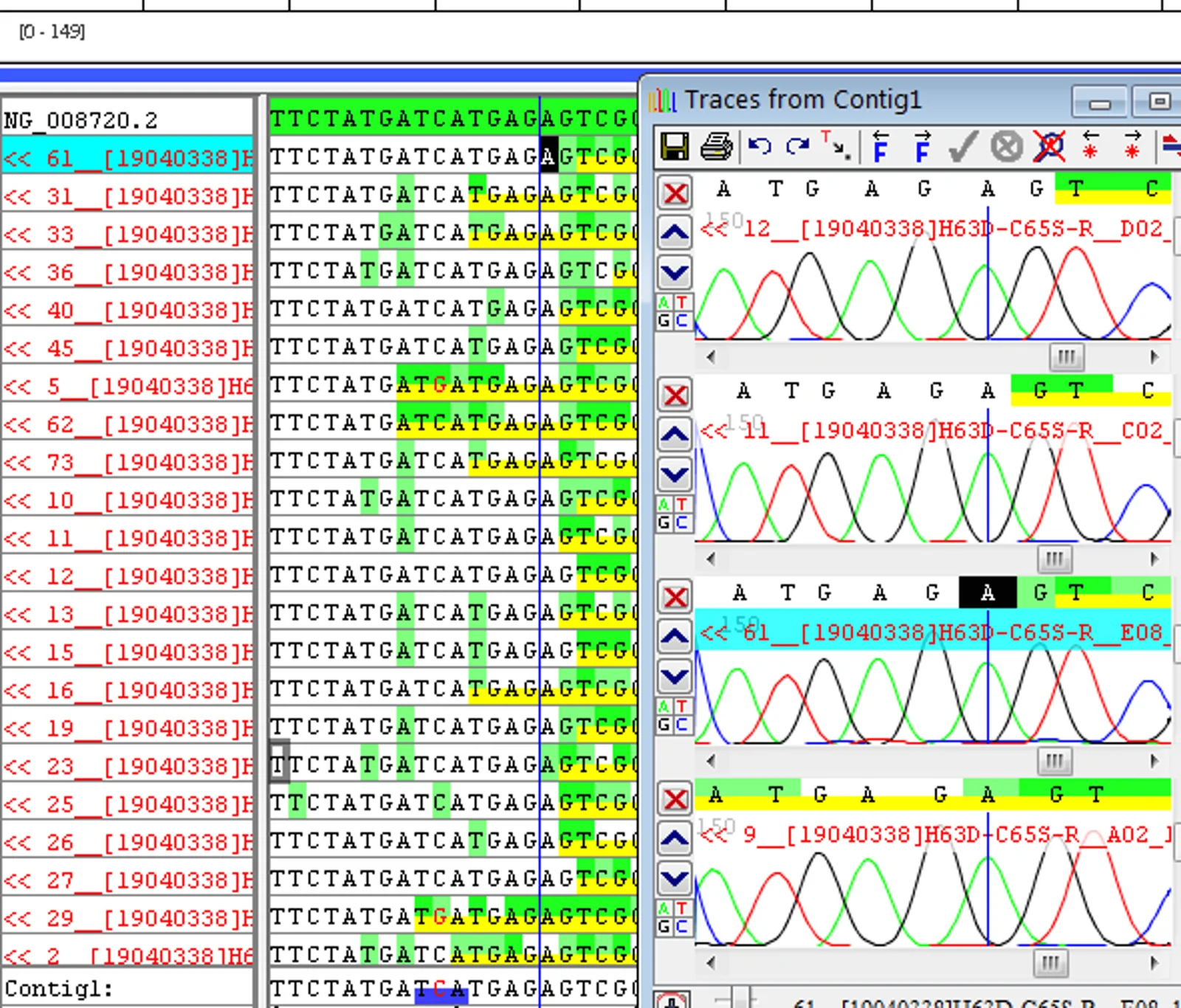
Chromatogram of S65C mutation The middle alignment showed that adenine (highlighted in black) was not substituted by thymine. The chromatogram on the right showed a single green adenine peak, indicating a wild-type allele in all patients.

Sequencing of the amplified *HFE* gene fragment for the S65C mutation (rs1800730) revealed only the wild-type allele (A) in all the participants (Figure 4).

## Discussion

Progressive iron accumulation is a critical complication of βTM and is considered the predominant cause of advanced morbidity and mortality in these patients [5]. This study aimed to screen Sudanese children with βTM for *HFE* gene H63D and S65C mutations to evaluate their impact on the severity of secondary IO.

A total of 76 children with βTM were enrolled in this study; all were receiving regular blood transfusions and iron chelation therapy and were monitored at various hospitals and health centers in Khartoum State, Sudan.

Spearman's rank correlation analysis demonstrated a statistically significant positive correlation between SF levels and the total number of blood transfusions (p = 0.496, p < 0.001). This supports the well-established observation that transfusional iron loading remains the main cause of elevated ferritin in transfusion-dependent β-thalassemia, as each transfusion adds a significant amount of iron that accumulates over time [18]. Although only a weak, non-significant positive correlation was observed between SF levels and the interval since the last iron chelation therapy dose (ρ = 0.257, p = 0.100), the finding remains clinically relevant, as effective iron chelation therapy depends on long-term adherence and persistence. Ensuring adherence to chelation therapy is particularly challenging because the benefits of treatment are not immediately perceived by patients. Consequently, poor adherence can substantially compromise treatment effectiveness, leading to inadequate control of IO and an increased risk of iron-related complications [19]. The wide variability in the interval since the last chelation dose observed in the present study (mean ± SD: 192 ± 231 days) suggests inconsistent adherence to treatment among some patients. This observation is consistent with previous reports indicating that maintaining compliance with chelation therapy is a major challenge in transfusion-dependent β-thalassemia, particularly in developing countries [20].

The lack of a significant correlation between SF and CRP levels among participants in this study indicates that inflammation did not significantly affect ferritin levels in our population and that SF levels primarily reflect iron burden rather than an inflammatory response.

In the present study, the *HFE* H63D mutation was identified in 17 patients (22.4%): one (1.3%) was homozygous and 16 (21.1%) were heterozygous, suggesting a relatively high frequency. The frequency of the wild-type allele (C) was 88%, and that of the mutant allele (G) was 12%. Comparable frequencies of this mutation have been documented in several regional studies. In Egypt, the heterozygous H63D mutation was reported in 25.4% of βTM patients [21]. Similarly, a study in Turkey reported a carrier frequency of 18.18% [22]. An inconsistent finding was reported in India, where no H63D mutations in the *HFE* gene were detected [23]. This variation in mutation frequency indicates complex regional and ethnic differences in the frequency of the H63D mutation.

Patients with the H63D mutation had higher SF levels than those with the wild-type allele, but the difference was not statistically significant. This suggests that the H63D mutation might not significantly affect the severity of secondary IO in Sudanese patients with βTM. This finding agrees with many previous studies reporting no significant association between the H63D variant and increased iron in βTM [24-26]. We suggest that the substantial transfusion-related iron burden, along with the chelation therapy in βTM, may obscure the partial loss of *HFE* protein function caused by this mutation and, thus, its effect on iron metabolism. 

After excluding patients with elevated CRP (N = 34) to minimize the confounding effect of inflammation, the association between the H63D mutation and IO severity remained not statistically significant. This finding further supports our earlier finding that inflammation does not contribute to elevated SF in our study population and that regular transfusions are the main factor.

Most patients with the heterozygous mutant genotype in this study had SF levels below 2500 µg/L, a threshold often associated with a higher risk of myocardial iron loading [27]. This result further supports our finding that this mutation has an insignificant effect on IO severity and aligns with two previous studies. The first study, by Fekri et al., examined the effect of *HFE* gene polymorphisms on ferritin levels in patients with βTM and reported no significant correlation between H63D polymorphism and blood ferritin levels [28]. The second, by Gochee et al., concluded that the presence of the H63D mutation does not result in significant IO in patients with β-thalassemia [29]. However, some evidence contradicts this view and suggests that the H63D mutation plays a more significant role in worsening iron loading in βTM, particularly affecting SF and alanine aminotransferase levels, while not significantly altering other biochemical or hematological parameters [17].

In the current study, the single patient with a homozygous H63D mutation had a markedly elevated SF level (4022 µg/L). This aligns with the findings of Longo et al., who also found a single patient with H63D homozygosity, and he was severely iron-loaded [30].

None of the thalassemic children in this study carried the *HFE* S65C mutation. This agrees with a study in Turkey by Turedi et al., which examined *HFE* polymorphisms, including S65C, and their association with cardiac iron loading in patients with βTM and reported the absence of all studied polymorphisms except H63D [22].

From a clinical perspective, our findings suggest that routine screening for *HFE* H63D and S65C mutations in all Sudanese children with βTM is not currently justified. Although H63D was relatively frequent, it was not significantly associated with higher SF levels or an increased risk of myocardial iron loading, and only a single homozygous case had markedly elevated ferritin levels. These observations, however, should be interpreted with caution given the limited sample size. Given the observed group sizes of 59 participants with the wild-type genotype (CC) and 17 H63D variant-allele carriers (CG + GG), the minimum detectable effect size was estimated using the normal approximation to the Mann-Whitney U test, assuming a two-sided α of 0.05 and 80% statistical power. The available sample could detect an effect corresponding to an approximate probability of superiority of 0.72 (rank-biserial correlation ≈ 0.45). Therefore, the study primarily detected moderate-to-large differences in SF levels between genotype groups, whereas smaller genotype-related effects may have gone undetected.

The major limitation of this study was the relatively small sample size, which limited the statistical power to detect small genotype-phenotype associations and may have masked subtle effects of *HFE* variants. Additionally, IO was assessed primarily using SF levels. Although SF is widely used as a practical and accessible marker of body iron stores, its levels may be influenced by multiple factors, which limits its accuracy as a sole measure of iron burden. Additional measures of IO were not available. Future studies involving larger cohorts and comprehensive IO assessment methods are needed to confirm these findings and further evaluate the clinical significance of *HFE* variants in patients with βTM.

## Conclusions

The *HFE* H63D mutation was relatively common among Sudanese children with βTM, occurring in 22.4% of patients, whereas the S65C mutation was not detected in any participant. Despite its relatively high frequency, the H63D variant was not significantly associated with higher SF levels, even after excluding patients with elevated CRP to minimize the confounding effect of inflammation. Although the single patient with homozygous H63D mutation exhibited markedly elevated ferritin levels, the rarity of this genotype precludes definitive conclusions regarding its clinical significance. These findings indicate that progressive IO in Sudanese children with βTM is primarily driven by cumulative transfusional iron loading and challenges in maintaining consistent adherence to iron chelation therapy, rather than by the presence of *HFE* gene mutations.

## References

[1] Langhi D Jr, Ubiali EM, Marques JF Jr, Verissimo MA, Loggetto SR, Silvinato A, Bernardo WM (2016). Guidelines on beta-thalassemia major - regular Blood Transfusion Therapy: Associação Brasileira de Hematologia, Hemoterapia e Terapia Celular: Project Guidelines: Associação Médica Brasileira - 2016. Rev Bras Hematol Hemoter.

[2] Kattamis A, Forni GL, Aydinok Y, Viprakasit V (2020). Changing patterns in the epidemiology of β-thalassemia. Eur J Haematol.

[3] Taher AT, Musallam KM, Karimi M, Cappellini MD (2012). Contemporary approaches to treatment of beta-thalassemia intermedia. Blood Rev.

[4] Mishra AK, Tiwari A (2013). Iron overload in beta thalassaemia major and intermedia patients. Maedica (Bucur).

[5] Shah S, Farooq N, Basharat A, Shah M, Mukhtiar S, Farhad S (2019). Frequency of iron overload complications in beta-thalassemia major patients. J Postgrad Med Inst.

[6] Kowdley KV, Brown KE, Ahn J, Sundaram V (2019). ACG clinical guideline: hereditary hemochromatosis. Am J Gastroenterol.

[7] Jallow MW, Cerami C, Clark TG, Prentice AM, Campino S (2020). Differences in the frequency of genetic variants associated with iron imbalance among global populations. PLoS One.

[8] Katsarou MS, Papasavva M, Latsi R, Drakoulis N (2019). Hemochromatosis: hereditary hemochromatosis and HFE gene. Vitam Horm.

[9] Gerhard GS, Paynton BV, DiStefano JK (2018). Identification of genes for hereditary hemochromatosis. Methods Mol Biol.

[10] Trifa AP, Popp RA, Militaru MS (2012). HFE gene C282Y, H63D and S65C mutations frequency in the Transylvania region, Romania. J Gastrointestin Liver Dis.

[11] Leão GD, Freire JM, Cunha Fernandes AL (2014). Analysis of HFE genes C282Y, H63D, and S65D in patients with hyperferritinemia from northeastern Brazil. J Clin Lab Anal.

[12] Silva B, Faustino P (2015). An overview of molecular basis of iron metabolism regulation and the associated pathologies. Biochim Biophys Acta.

[13] Ekanayake D, Roddick C, Powell LW (2015). Recent advances in hemochromatosis: a 2015 update: a summary of proceedings of the 2014 conference held under the auspices of hemochromatosis Australia. Hepatol Int.

[14] Salgia RJ, Brown K (2015). Diagnosis and management of hereditary hemochromatosis. Clin Liver Dis.

[15] Wilson MM, Al-Wakeel H, Said F, El-Ghamrawy M, Assaad M, El-Beshlawy A (2015). Study of the effect of HFE gene mutations on iron overload in Egyptian thalassemia patients. Egypt J Med Hum Genet.

[16] Zekavat OR, Zareian Jahromi M, Haghpanah S, Kargar Jahromi Z, Cohan N (2021). Association of HFE gene mutations with serum ferritin level and heart and liver iron overload in patients with transfusion-dependent beta-thalassemia. J Pediatr Hematol Oncol.

[17] Maatooq SA, Alwash MM, Ahmed AA (2022). Comparison between H63D and G71D gene mutation effects on iron overload in Iraqi patients with β-thalassemia major: a case-control study. Iraqi J Hematol.

[18] Nandi S, Samanta S, Mondal T, Halder S (2021). Study of lipid profile in β thalassemia major pediatric patients with multiple blood transfusion and its correlation with serum ferritin level in tertiary care hospital in Kolkata. Int J Community Med Public Health.

[19] Locke M, Reddy PS, Badawy SM (2022). Adherence to iron chelation therapy among adults with thalassemia: a systematic review. Hemoglobin.

[20] Chong CC, Redzuan AM, Sathar J, Makmor-Bakry M (2021). Patient perspective on iron chelation therapy: barriers and facilitators of medication adherence. J Patient Exp.

[21] El-Rashidi FH, Elshafey AE, Ragab SM, Hegazy FF, El-Nabi SEH (2008). Haemochromatosis gene mutation H63D is a risk factor for iron overload in Egyptian beta- thalassemic children. Egypt J Med Hum Genet.

[22] Turedi A, Oymak Y, Meşe T (2013). The effect of HFE polymorphisms on cardiac iron overload in patients with beta-thalassemia major. Pediatr Hematol Oncol.

[23] Iyer N, Kulkarni A, Patel J, Chuhan J, Singh K (2014). Study of HFE gene mutation at C282Y and H63D locus with special reference to thalassemia patients. Int J Agric Environ Biotechnol.

[24] Politou M, Kalotychou V, Pissia M, Rombos Y, Sakellaropoulos N, Papanikolaou G (2004). The impact of the mutations of the HFE gene and of the SLC11A3 gene on iron overload in Greek thalassemia intermedia and beta(s)/beta(thal) anemia patients. Haematologica.

[25] Mellouli F, El Borgi W, Kaabi H (2006). HFE gene mutations in Tunisian major beta-Thalassemia and iron overload (Article in French). Transfus Clin Biol.

[26] López-Escribano H, Ferragut JF, Parera MM, Guix P, Castro JA, Ramon MM, Picornell A (2012). Effect of co-inheritance of β-thalassemia and hemochromatosis mutations on iron overload. Hemoglobin.

[27] Pennell DJ, Udelson JE, Arai AE (2013). Cardiovascular function and treatment in β-thalassemia major: a consensus statement from the American Heart Association. Circulation.

[28] Fekri K, Asle Rasouli N, Tavallai Zavareh SA, Jalil M, Moradi F, Hosseinpour M, Teimori H (2019). Hepcidin and HFE polymorphisms and ferritin level in β-thalassemia major. Int J Hematol Oncol Stem Cell Res.

[29] Gochee PA, Powell LW, Cullen DJ, Du Sart D, Rossi E, Olynyk JK (2002). A population-based study of the biochemical and clinical expression of the H63D hemochromatosis mutation. Gastroenterology.

[30] Longo F, Zecchina G, Sbaiz L, Fischer R, Piga A, Camaschella C (1999). The influence of hemochromatosis mutations on iron overload of thalassemia major. Haematologica.

